# Electrosurgery in aesthetic and restorative dentistry: A literature review and case reports

**DOI:** 10.4103/0972-0707.58332

**Published:** 2009

**Authors:** Kusum Bashetty, Gururaj Nadig, Sandhya Kapoor

**Affiliations:** Department of Conservative Dentistry and Endodontics, The Oxford Dental College and Hospital, Bommanhalli, Hosur Road, Bangalore-560 068, Karnataka, India

**Keywords:** Bipolar electrosurgery, crown lengthening, electrosurgery, gingivectomy, monopolar electrosurgery

## Abstract

Electrosurgery has been used in dentistry for more than half a century. There is abundant literature on electrosurgery dating back more than a century. During the past three decades, a substantial increase in minimally invasive surgery and microvascular surgery prompted greater use of electrosurgery. Although this surge in utilization has resulted in new applications, equipment features, problems and solutions, the use of electrosurgery in the field of restorative dentistry has remained relatively unchanged. The presence of conflicting and sometimes confusing information on electrosurgical wound healing in the dental literature is the most likely reason. This article briefly explains the literature review of electrosurgery and clinical application of electrosurgery in aesthetic and restorative dentistry.

## INTRODUCTION

The interface between dental restorations and the surrounding soft tissue is of critical importance for restorative success.[1] An individual tooth is aesthetically inseparable from the adjacent gingiva. Successful aesthetic and restorative dentistry can be best accomplished when healthy and stable tissues surround the teeth. Close attention to both soft and hard tissues around teeth before, during and after restorative procedures will greatly increases the probability of a successful outcome.

Gingival overgrowth is a condition that commonly develops after trauma or is associated with cervically fractured teeth. Gingiva can be surgically reshaped and recontored for cosmetic, physiological or functional purposes and is referred to as crown lengthening procedure. Crown lengthening is a periodontal resective procedure aimed at removing supporting periodontal structures to gain sound tooth structure above the alveolar crest level. Gingivoplasty corrects the remaining thick and un-naturally shaped gingival tissue left after the gingivectomy procedure and exposes the hidden teeth.

Three methods of cutting oral soft tissue in dentistry are scalpel, electrosurgery (ES) and laser. Each of these methods works. However, they are different from the standpoints of hemostasis, healing time, cost of instruments, width of the cut, anesthetic required and disagreeable characteristics, such as smoke production, odor of burning flesh and undesirable taste.

Cutting soft tissue with a scalpel is a technique used by every dentist. All practitioners know the negative characteristics associated with cutting soft tissue with a scalpel, including excessive blood flow and inadequate visibility caused by blood in the operating field. However, dentists also know that there are desirable aspects of using a scalpel for soft-tissue cutting, including ease of use, low cost and relatively fast and uneventful healing.

Since 1914, ES has been used routinely in various aspects of medicine, including dentistry. ES has been defined as the intentional passage of high-frequency waveforms or currents, through the tissues of the body to achieve a controllable surgical effect.[2] By varying the mode of application of this type of current, the clinician can use ES for cutting or coagulating soft tissues. The ES equipment, if used for such procedures, minimizes bleeding and most patients experience very little post-operative pain after the procedure. Since the introduction of lasers in dentistry, use of ES has further declined. A comparison of laser with ES reveals significant overlap of potential uses and effectiveness. Both laser and ES units work well for simple cutting of oral soft tissues.

This article presents a literature review of ES and case reports where ES is used for cutting gingival soft tissue. ES is simple, cost effective and yield good results, along with good patient satisfaction.

## ELECTROSURGICAL CURRENTS ON TISSUE: CUTTING AND COAGULATION

Electrosurgical therapeutic objectives pertinent to restorative dentistry include cutting, which is principle use of ES, and coagulation. A review of the effects of electrosurgical currents on tissue is important for comparing the two modes.

Alternating current changes direction regularly from positive to negative (waveform), with the number of wave units (cycles per seconds) establishing the frequency (1 cycle = 1 Hz). Anions and cations in cells attain to align themselves with the polarity of the current. The rapidity of the alternating polarity in ES (300 KHz to 4 MHz), however, does not allow alignment but rather rapid oscillation of the ions. Oscillation creates friction and is one means of converting electrical energy to thermal energy in tissue. This process is referred to as the dielectric effect (dielectric loss) and is related to the frequency of the alternating current.[3] Geddes considers dielectric loss to be a negligible component in the conversion of electrical energy to thermal energy and views the resistance to current flow by the tissue to be the most important component in the generation of heat. The flow of alternating current in tissue is complex and dynamic.

Texts on ES[4–6] provide the following outline of events when high-frequency current is applied to soft tissues:

1. As temperature of tissue increases to 45°C from a resting temperature of approximately 37°C, reversible changes occur that can be assessed only by chemical means.
2. As temperatures are sustained beyond 45°C, coagulation of the protein contents of cell occurs (white coagulation). It is an irreversible state. However, tissue or cellular form remains intact.
3. When tissue temperature rises above 60°C, the water content of the cell is driven out, and the process of desiccation begins. This process continues until all water is dissipated or until the temperature rises above 100°C. Desiccation type of coagulation is the therapeutic objective for accomplishing hemostasis.[5,7]
4. Beyond desiccation, continued application of heat causes disintegration of cellular components into oxygen, nitrogen, hydrogen and other elements including carbon.[5] This disintegration is observed clinically as an Escher, often described as black coagulation or carbonization.
5. If energy transfer occurs at a higher intensity and rate, vaporization occurs. When temperature rapidly exceeds 100°C, the vapors generated within the cell cannot be contained, and the cell virtually explodes. Under these circumstances, temperature can rise to 400°C to 600°C in microsecond intervals such that the cells are physically disrupted as the fluids vaporize. The sequence of events is so concentrated, intense and rapid that cells nearby are virtually unaffected. The mixture of cell particles and smoke is referred to as plume. This process describes the events in electrosection and fulguration.[5,6]

These steps describe a series of events in which the current is applied at an intensity level and over an interval of time, which allows each of the phases to occur.

## COMPARISON OF MONOPOLAR AND BIPOLAR SYSTEMS

There are two basic types of ES, monopolar and bipolar units.[4,8] In monopolar ES units, the current begins with the ES device and travels along a wire to the oral site, then to an indifferent plate placed behind the patient's back. As the surgical electrode contacts the patient's oral soft tissues, heat is produced and controlled cutting is achieved. Bipolar ES devices have two electrodes on the cutting tip. The current flows from one electrode to the other, making a broader cut than does the monopolar unit, but eliminating the need for the indifferent plate.

### Current frequency

Electrosurgical units typically operate at one fixed frequency predetermined by the manufacturer for each model. Multifrequency ES units are cost prohibitive. The impact of the choice of current frequency is greater in the monopolar than the bipolar mode and is more significant in electroincision than in electrocoagulation.

### Active and dispersive electrodes

In the monopolar system, a high-frequency current is delivered to the active electrode which is proportionately much smaller than the dispersive electrode. The patient effectively becomes part of the circuit such that the current travels from the active electrode, through the patient, to the dispersive electrode.

In bipolar systems, the dispersive electrode is typically the same size and shape as the active electrode, and both are typically attached to the same probe tip or handpiece.

### Dissipation of the current

In the bipolar mode, both electrodes are applied concurrently and the current travels from the active electrode through the limited intervening tissue directly to the dispersive electrode. Unlike the monopolar mode, the area of high-current density, the tissue serving as the return path and the tissue involved in the immediate diffusion zone are all virtually the same or within very narrow limits. These features enable the safe use of the bipolar mode on tissues with other vital structures in the vicinity.

### Path of the current

The path that the current takes from the active to the dispersive electrode in the monopolar mode is beyond the control of the operator since the current will independently find the least resistant path, which is not necessarily the shortest path. Nerves and vessels are more conductive than fat, bone, enamel, dentin and cementum. This limits the use of monopolar surgery near and around critical structures. Since current flow is limited to the tissue between the two electrodes in the bipolar mode, only the tissue, not major organs or a substantial portion of the patient, becomes part of the circuit.

Both types of electrosurgical units achieve their intended purposes well, but monopolar electrosurgery is used more often than is bipolar electrosurgery.

## WOUND HEALING IN ELECTROSURGERY

The histological effect of ES varies depending on the power output[9] and frequency[10] of ES unit, the waveform selected[11] and size and shape of active electrode.[12] In a comparative study of electrosurgical and scalpel wounds, it was observed that healing of electrosurgical wounds was delayed.[13] Electrosurgical wound had more inflammatory response and more tissue destruction. But in both kinds of wounds the viability of osteoblasts was the same, and there was no increase in the osteoclasts which would indicate that no bone resorption had occurred.[13] On the contrary, Glickman and Imber[14] found that there was no difference on wound healing between ES and periodontal knives when the gingival resection was shallow. In deep resection however they found intense inflammation and loss of bone height resulting from bone necrosis. Another study[15] showed that although there is loss of tissue soon after ES, 70 to 100% of the lost tissue is regained over a period of months. When teeth with cervical amalgam restoration were contacted by active electrode tip to simulate clinical application, it was found that contrary to an earlier report,[16] there was no evidence of extensive damage or necrosis of pulp.[17]

In a review on healing of electrosurgical wounds, Williams[18] has shown that many of the reports on electrosurgical wound healing have not mentioned the type of ES unit, the waveform, size and shape of electrode used, nor the speed at which electrode was passed over the tissue. Therefore, it is not possible to know whether some of the delayed wound healing was reported was the results of the operators' not having an optimal control of the factors involved in ES or whether ES is actually as damaging as it has been portrayed.

## APPLICATIONS OF ELECTROSURGERY IN AESTHETIC RESTORATIVE DENTISTRY

The therapeutic objectives desired in restorative dentistry include incision (or excision) and coagulation of tissue. Both monopolar and bipolar modes can achieve cutting and coagulation of tissue. The monopolar mode, however, is more effective than bipolar for cutting action and possesses distinct advantages over the bipolar mode for this objective.[4] A wide variety of electrode shapes is available for monopolar cutting than is available in the bipolar mode. Although cutting can be accomplished by the bipolar mode, it is much more inefficient and restrictive in application. Bipolar coagulation is slower than monopolar activity.[4] The slower action is considered a disadvantage for surgical procedures but proves to be a significant benefit for the more precise microsurgical procedures. Reports of mucosal and osseous necrosis[19–22] have substantially stifled the use of ES, particularly in the periodontal sector. It should be noted that these complications were exclusively associated with monopolar ES. This article presents certain case reports carried out using monopolar electrosurgical unit with good post operative results.

## CASE REPORTS

### Case 1: Gingival recontoring

**Figure 1:**
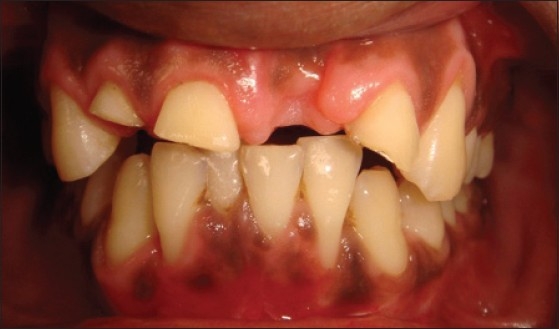
Pre-operative picture shows fracture of teeth and gingival overgrowth

**Figure 2:**
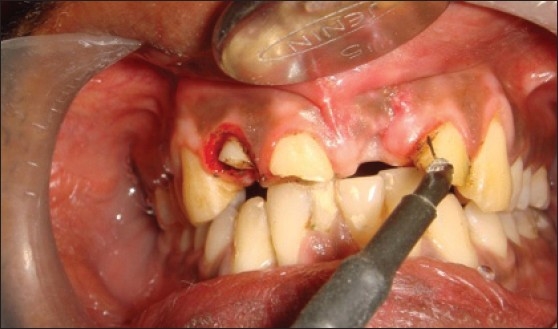
Use of electrosurgical electrode for gingivectomy

**Figure 3:**
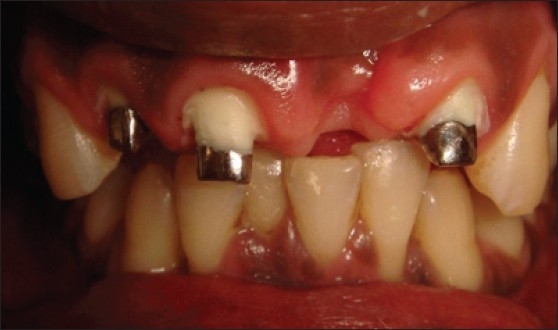
Custom-made post and core

**Figure 4:**
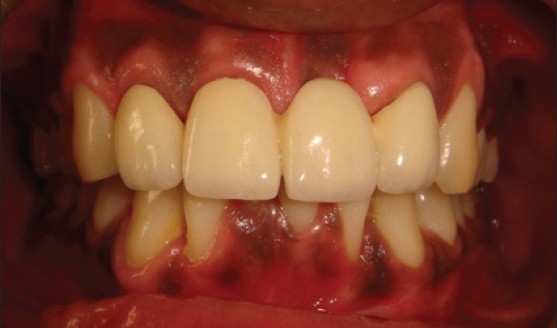
PFM bridge from right to left lateral incisor

Patient presented with fracture of right central (11), lateral (12) and left lateral incisor (22). Left central incisor (21) was missing. Vitality test showed negative response with all the three teeth. Gingival overgrowth was present in relation to all the three teeth on labial and palatal aspect [Figure 1]. Root canal treatment was carried out for all the three teeth followed by removal of excessive soft tissue on labial and palatal aspect using electrosurgery unit [Figure 2]. Patient was recalled after three days for post and core procedure [Figure 3]. Postoperative healing of the gingival tissue and placement of PFM bridge from right to left, lateral incisor [Figure 4].

### Case 2: Excision of gingival tissue extending into carious lesion

**Figure 5:**
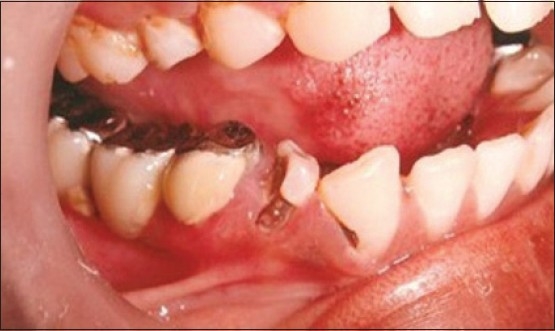
Presence of caries and gingival overgrowth

**Figure 6:**
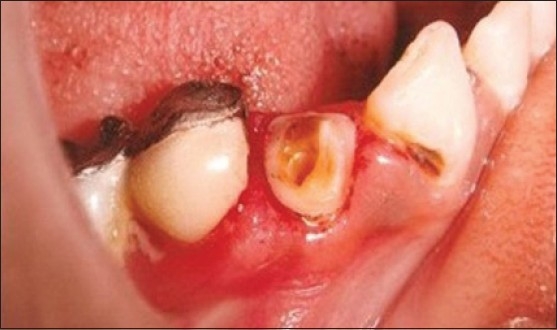
Gingivectomy using ES

**Figure 7:**
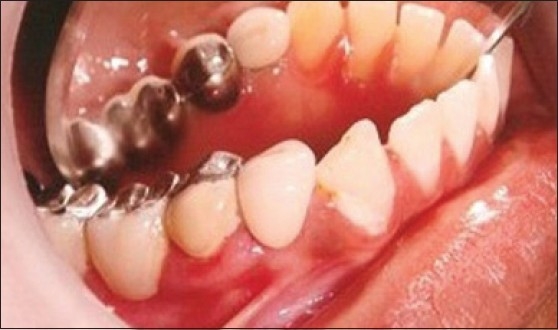
Post operative photograph showing PFM crown with right lower premolar

Patient presented with caries on right lower canine (43) and premolar (44). Overgrowth of gingiva was present into the carious lesion and interproximally between 44 and 45 [Figure 5]. Excision of gingival overgrowth was carried out using electrosurgery [Figure 6]. Root canal treatment followed by post and core and crown [Figure 7].

### Case 3: Excision of gingival tissue extending into fractured area of the tooth

**Figure 8:**
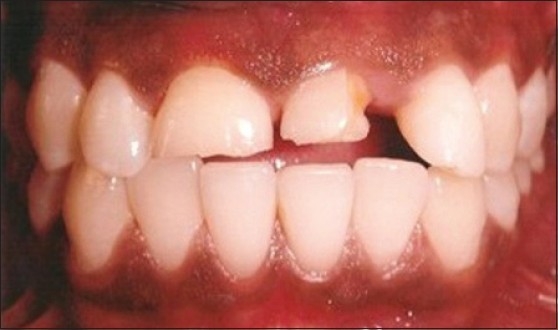
Fracture of right and left central incisors

**Figure 9:**
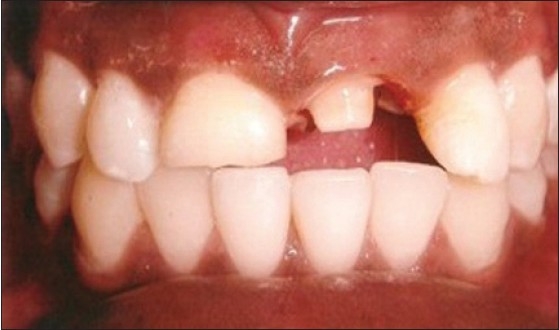
Use of ES distal to left central incisor

**Figure 10:**
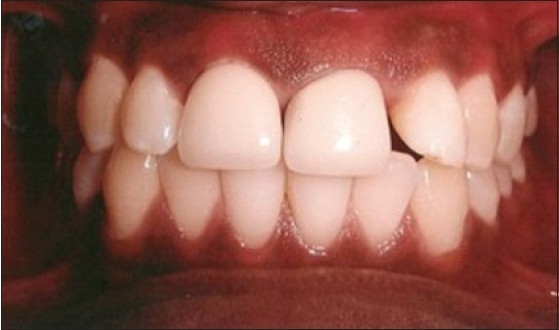
PFM crown with right and left central incisors

Patient presented with fracture of right (11) and left central incisor (21) with gingival overgrowth present interproximally distal to 21 [Figure 8]. Vitality test showed negative response in relation to 21. Root canal treatment followed by crown preparation was done in relation 21 and gingival tissue was removed using electrosurgery [Figure 9]. On 2^nd^ appointment, crown preparation was done in relation to 11 and post and core cementation was done in relation to 21. After four days PFM crown was cemented [Figure 10].

## CONCLUSION

ES has several applications in almost all branches of dentistry, but this technique is not very widely used. ES has always had serious limitations in the oral cavity. Some problems included its inability to touch bone or metal, high heat and current spread, the need for a grounding pad, and the fact that it cuts by an advancing spark. These limitations have limited its acceptance by many general dentists and almost a complete avoidance by periodontists and oral surgeons. Regular users of ES know from experience, that when ES is applied according to principles, predictable and good wound healing can be achieved. ES can never completely replace the scalpel but although ES requires more knowledge and skill, the advantages out number and outweighs its disadvantages. An electrosurgical unit costs only a small fraction of the price of laser unit and can be used to perform many of the soft tissue surgical procedure, being carried out with laser. If the clinician practices ES techniques in the laboratory and applies them clinically according to the principles, clinician will surely find ES to be of immense use in clinical dentistry.
